# Regional Anesthesia in Trauma Medicine

**DOI:** 10.1155/2011/713281

**Published:** 2011-11-21

**Authors:** Janice J. Wu, Loreto Lollo, Andreas Grabinsky

**Affiliations:** Department of Anesthesiology and Pain Medicine, Harborview Medical Center, University of Washington, #359724, 325 Ninth Avenue, Seattle, WA 98104, USA

## Abstract

Regional anesthesia is an established method to provide analgesia for patients in the operating room and during the postoperative phase. While regional anesthesia offers unique advantages, as shown by the recent military experience, it is not commonly utilized in the prehospital or emergency department setting. Most often, regional anesthesia techniques for traumatized patients are first utilized in the operating room for procedural anesthesia or for postoperative pain control. While infiltration or single nerve block procedures are often used by surgeons or emergency medicine physicians in the preoperative phase, more advanced techniques such as plexus block procedures or regional catheter placements are more commonly performed by anesthesiologists for surgery or postoperative pain control. These regional techniques offer advantages over intravenous anesthesia, not just in the perioperative phase but also in the acute phase of traumatized patients and during the initial transport of injured patients. Anesthesiologists have extensive experience with regional techniques and are able to introduce regional anesthesia into settings outside the operating room and in the early treatment phases of trauma patients.

## 1. Background

Compressing peripheral nerves over an extended period of time to cause profound analgesia distal to the site of compression is a historical method of regional anesthesia described in the 16th century by the French military surgeon Ambroise Par*ē* (1510–1590). Dominique Jean Larrey (1766–1842), surgeon-in-chief in Napoleon's army, described his observation of cold injury on nerve function and its analgesic effect on soldiers during amputations.

The anesthetic properties of cocaine were known and published in the 19th century. In 1984, Carl Koller (162–1944) recognized the importance of these findings and instilled an aqueous solution of cocaine onto the cornea of a frog. He presented this experiment at the German Ophthalmological Society meeting in Heidelberg later that year. In the following years, most of the regional anesthesia techniques were developed and continue to be used today, much as they were in those years. The brachial plexus block under surgical exposure using cocaine was first performed in 1884 by Crile [1]. The first percutaneous block was described in 1911 by Hirschel and in the same year by Kuflenkampf [2, 3]. In 1884, the same year Koller presented his findings, Corning performed the first epidural anesthesia, and published the procedure in the New York Medical Journal in 1885 [4]. In 1898, Bier (1861–1949) and his resident Hildebrand (1868–1954) performed the first spinal anesthesia and published their personal experiences after they attempted spinal anesthesia on each other. While Hildebrand had good analgesia from the spinal anesthesia, both physicians had severe headaches and Bier discouraged the use of spinal anesthesia and it took several more years before spinal anesthesia became an established regional technique [5]. In 1908, Bier described the intravenous injection of local anesthetics, called the Bier block [6]. Many of the early techniques are still in use today and are often only supplemented by newer techniques or medications. 

Newer regional anesthetics allow the use of long- or short-acting drugs depending on the desired length of pain relief. The introduction of specific regional nerve block needles and catheters in the late 19th and early 20th century and more recent technical advances including nerve stimulation and ultrasound guidance, have helped to advance the practice of regional anesthesia and to improve the precision and safety of peripheral nerve blocks and neuraxial procedures for patients with acute pain [7]. 

Regional anesthesia techniques offer excellent pain control and are commonly utilized during surgery and in the postoperative phase, thus decreasing the amount of anesthetics and intravenous analgesics used for pain control. In addition, outcome studies have shown that regional anesthetic techniques can hasten recovery, decrease ICU and hospital length of stay, improve cardiac and pulmonary function, decrease infection rates and neuroendocrine stress responses, and promote earlier return of bowel function [8]. 

Regional techniques provide not only excellent analgesia, but the absence of systemic sedation makes it easier to monitor the mental status of patients with head injuries. Despite these known benefits, regional anesthetic techniques have been underutilized in trauma patients, especially during the acute phase of injury [9]. One study reports that up to 36% of patients with acute hip fractures in the emergency department received no analgesia and even fewer patients were considered for regional nerve blocks [10, 11]. Compared to an elective surgical patient, where analgesia needs are addressed during the peri-operative period, the trauma patient during the acute phase requires constant assessment and treatment of pain from the acute prehospital or battlefield environment, during transport to the emergency room, and during subsequent care in the operating room and intensive care unit. The stress and inflammatory responses following acute trauma are even greater than those experienced with elective surgery [8]. In addition, trauma patients vary in the extent and number of injuries sustained and these can have a variable effect on their mental status, respiration, and hemodynamic stability, all of which can be exacerbated by parenteral analgesics. The experience of caring for injured soldiers during recent military conflicts has led to unique approaches employing regional anesthetic techniques for field analgesia and surgical anesthesia [7, 12, 13]. Anesthesiologists have assumed a vital role in the care of these patients and, together with surgeons and emergency medicine physicians, have introduced methods to provide safe and timely treatment and transport of injured soldiers. These experiences, along with outcome studies looking at the early utilization of nerve blocks in the emergency department [10, 11, 14] illustrate the benefits of regional analgesia compared to traditionally used intravenous opioid regimens in acutely injured patients and during transport.

## 2. Regional Analgesia in the Early Phase of Trauma

One of the advantages of early utilization of regional anesthesia is to reduce intravenous opioid requirements in order to adequately relieve pain. A functioning peripheral nerve block, using a long-acting local anesthetic with fast onset time, attenuates the stress response to injury, and reduces the incidence of untoward dose-related opioid side effects including respiratory depression, increased sedation, confusion, pruritus, and nausea [8]. Additional benefits demonstrated in patients receiving peripheral nerve blocks in the pre-hospital setting include safer transport and a decreased need for their medical supervision and in the setting of mass casualties, a stable, comfortable, and awake patient allows for decreased staffing [11]. 

Recent literature from the battlefield suggests that the use of regional anesthesia as an early intervention improves safety and reduces pain and injury-related complications. In addition to the short-term benefits of acute pain control, early treatment of injuries to the extremities has potential long-term benefits including reduction in the incidence and severity of chronic pain sequelae such as causalgia and posttraumatic stress disorder [13]. When translating the use of regional techniques for pain control used preoperatively to techniques feasible for prehospital providers or the emergency room, it is important to evaluate the equipment and staffing available in these settings. 

Not all regional techniques are equally suitable for the prehospital setting or the emergency room and not all providers are equally trained or experienced in regional techniques. Especially, neuraxial techniques such as continuous thoracic epidural catheters, as commonly utilized for abdominal procedures or rib fractures, can result in significant complications such as hypotension and spinal cord injury. The use of these techniques depends very much on the available expertise and staffing model in the emergency room.

Extremity blocks on the other side are often easy to perform, even without ultrasound or nerve stimulation, and have a lower risk of hypotension or complications. 

Rib fractures and lower-extremity fractures are commonly encountered in the emergency department. These injury patterns are also quite accessible for easy-to-perform regional techniques. Several studies have compared regional anesthesia techniques with the more traditional opioid administration in the emergency department and in the early stages of hospitalization. Feasibility of continuous nerve block catheters for long-term infusion has also been investigated.

### 2.1. Hip and Lower Extremity Injuries

Buckenmaier et al. illustrated the value of peripheral nerve blocks for both prolonged pain management and repeated surgical interventions in a report on the placement of continuous lumbar plexus and sciatic nerve catheters in a soldier shortly after sustaining a lower-extremity injury in the battlefield. The ability to provide anesthetic and analgesic doses of local anesthetics via lumbar and sciatic catheters during his evacuation and throughout his hospitalization for 16 days was site specific, reliable for pain control, and avoided the risks associated with exposure to high doses of opioids, general anesthesia, and repeated nerve blocks [12]. Despite the eventual need for amputation, the patient did not develop phantom limb pain or other chronic pain syndromes. 

Prior to the more recent military experiences, European authors described single nerve block procedures performed in the field by pre-hospital emergency physicians and anesthesiologists at the scene of the accident and during transport. A single injection femoral nerve block performed at the scene in elderly patients with knee pain after trauma has been shown to provide effective analgesia and facilitate transport. Barker et al. studied the effect of a single-shot femoral nerve block compared to intravenous analgesia with metamizole given prior to hospitalization. This randomized control trial demonstrated that the femoral nerve block promoted earlier reduction of pain and attenuated the sympathetic stress response. Furthermore, in experienced hands, the femoral nerve block was shown to be a safe technique that was easy to perform and caused minimal delays in transport [11]. Given the safety and ease of identifying the anatomical landmarks surrounding the femoral nerve, several studies have looked at the utility of femoral nerve or fascia iliaca compartment blocks in the emergency room. Both these blocks are easy to perform and have been effective in providing pain relief for femoral neck fractures and hip fractures. 

The acute pain associated with femoral fractures has been described to be excruciating and one of the more painful fractures [15]. The use of peripheral nerve blocks in this patient population has been shown to improve analgesia more rapidly and increased patient satisfaction compared to parenteral and intramuscular opioid administration [14, 16]. Mutty et al. demonstrated that a femoral nerve block significantly reduces acute pain from distal femoral fracture when compared to IV opioids. Fifty-four patients were randomized to each treatment arm. Patients receiving a femoral nerve block had an average reduction in their pain scores of 3.6 points when compared to traditional management with intravenous hydromorphone. Results were observed as early as five minutes after intervention [16]. A similar study by Wathen et al. compared the effects of fascia iliaca compartment nerve block (FICB) to intravenous (i.v.) morphine in children presenting to the emergency department with an acute femoral fracture. In this controlled unblinded study, fifty-five patients were randomized to receive either an FICB or IV morphine. Patients who were in the FICB group had decreased pain scores at 30 min and 6 hrs after intervention, accompanied by fewer respiratory depression events, and a decreased incidence of muscle spasms [14]. In addition, satisfaction scores of physicians, nurses, parents, and patients were all higher in the FICB group. 

 These studies confirm the findings of earlier smaller-scale studies and reports on the efficacy of femoral nerve blockade. In both studies, orthopedic residents and emergency room physicians did the procedures, respectively, after initial training by an anesthesiologist. The promising results of single injections have lead to studies comparing single-shot injections to early catheter placement for continuous pain control during hospitalization. Stewart et al. describes femoral nerve block procedures performed by emergency medicine physicians, including continuous catheters in 40 pediatric patients with femoral fractures [17].

One of the limitations of these nonblinded studies is the potential for subjective bias from both the patient and providers. To further investigate, Foss et al. [10] designed a randomized double-blind placebo-controlled trial to compare the effect of FICB with standardized intramuscular (i.m.) morphine analgesia in patients with acute hip fractures. All forty-eight patients received an intragluteal injection and a fascia iliaca “block.” The FICB group received 1% mepivacaine with epinephrine for the FICB with an IM injection of saline while the morphine group received an IM injection of 0.1 mg/kg morphine and a saline FICB. The results of this study indicate that FICB provides superior pain relief both at rest and with dynamic movement of a 15 degree leg lift. In addition, the FICB, performed by anesthesiologists took on average 4 minutes. There were no reported side effects from the FICB while the morphine group had a tendency to lower oxygen saturation at 60 and 180 minutes, despite the use of supplemental oxygen.

### 2.2. Upper-Extremity and Should Injuries

Regional blocks of the brachial plexus for the upper-extremity surgeries are well established for preoperative pain relief. The brachial plexus can be blocked by different approaches, namely the axially, infraclavicular, and interscalene approach. Especially low-dose regional anesthesia potentially reduces the risk of local anesthetic toxicity and may be useful for procedures of short duration or lesser pain intensity such as procedures in the emergency room. In a prospective study, O'Donnell et al. compared low-dose ultrasound-guided axillary blocks with general anesthesia for patients undergoing upper-extremity surgery in the operating room. When compared with general anesthesia, patients receiving low-dose ultrasound-guided axillary blocks experienced excellent anesthesia, superior analgesia, reduction of opiate consumption, shorter recovery room times, and earlier hospital discharge [18]. 

Another common injury seen in the emergency room, is joint dislocations of the upper extremity, namely, elbow and shoulder dislocation. Shoulder dislocations, especially, often require deep sedation for reduction, when mild sedation does not allow the reduction due to muscle tension or pain control issues. Moderate or deep sedation requires the patient to be fasted and may prolong the patient's stay in the emergency room. The interscalene block offers excellent pain relief and muscle relaxation for this kind of procedure, as the shoulder is innervated by the superior and middle trunk close to the skin in the interscalene groove. A common failure of the interscalene nerve block, namely, not completely anesthetizing the inferior trunk formed by the C7 and T1 nerves, is not important for reduction of a dislocated shoulder. Blaivas et al. described 42 patients who received either sedation with etomidate or an ultrasound, guided interscalene block, performed by an emergency medicine physician. The length of stay (LOS) in the ED was significantly higher in the procedural sedation group (177.3 ± 37.9 min) than in regional group (100.3 ± 28.2 minutes). The mean (±SD) one-on-one healthcare provider time was 47.1 (±9.8) minutes for the sedation group and 5 (±0.7) minutes for the regional group. None of the receiving an interscalene blocks required any additional analgesia or sedation while performing shoulder reduction [19].

### 2.3. Rib Fractures

Rib fractures are a common injury associated with blunt trauma. They are associated with a significant amount of pain, and patients that presented with three or more fractured ribs have a higher risk of pulmonary complications. The pain can impair ventilation and ability to clear secretions, which can result in atelectasis and hypoxia. Up to 1/3 of patients develop nosocomial pneumonia, and mortality from isolated flail chest has been reported as high as 16%. Thus, general management goals include pain control, chest physiotherapy, and mobilization. The pain management guidelines for blunt thoracic trauma recommends epidural analgesia as the optimal and preferred modality for pain relief unless contraindicated. Placement of a thoracic epidural anesthetic in this situation results in a doubling of vital capacity in spontaneously ventilating patients, reduces paradoxical chest wall movement of the flail segments, and avoids the side effects of opioid narcotics including somnolence, respiratory depression, and gastrointestinal symptoms [20]. Bulger et al. demonstrated that thoracic epidural analgesia is associated with a decreased rate of nosocomial pneumonia and a shorter duration of mechanical ventilation. This prospective randomized trial included 458 blunt thoracic trauma patients. In patients with greater than three rib fractures, the epidural analgesia group had an average of 7.6 ventilator days compared to 9.1 days in the systemic opioid group. When adjusted for type of pulmonary injury, the risk of pneumonia in the systemic opioid group was six-times that of the epidural group. Despite these advantages, only 22% of patients were offered an epidural analgesia, with infection, coagulopathy, spinal fractures, and hemodynamic instability being the most common reasons for exclusion [21]. Alternatives to thoracic epidural anesthesia include paravertebral nerve blocks, intercostal nerve injections, and intrapleural catheters. Of these options, the paravertebral nerve block seems to be the most promising, although its efficacy has not been widely investigated.

## 3. Limitations of Regional Techniques

The disadvantages of regional analgesia are technical complexity of the procedure and the training and repetition required to achieve and maintain proficiency in regional techniques. Regional anesthesia is an invasive procedure with risks of infection, nerve injury, and procedure-specific risks such as vascular injury, pneumothorax, local anesthetic toxicity, infection, or possibly masking a compartment syndrome in extremity injuries. While for some patients with extensive extremity injuries multiple continuous catheter techniques can be utilized, often these patients require systemic analgesics and sedation, which may be more reasonable than regional techniques in some instances. 

Despite the benefits of regional analgesia, the utilization of these techniques is often either not considered or is deemed unsuitable due to the potential risks or side effects. Yet more often it is due to the lack of training or simply to the lack of knowledge about regional techniques by the medical staff treating these patient in the prehospital and emergency room phase. 

### 3.1. Compartment Syndrome

Trauma to the extremities can result in compartment syndrome where swelling and the increased tissue pressure in muscle compartments can reduce the circulation, resulting in ischemia and extensive muscle necrosis. One of the symptoms of compartment syndrome is increased pain. Even while increased pain is an unreliable symptom, it is thought that postoperative pain control, especially regional anesthesia may mask this symptom and result in the delay of diagnosis. A delay in the diagnosis and treatment of compartment syndrome, following orthopedic injury to the long bones can result in disastrous outcomes including amputation, renal failure resulting from rhabdomyolysis, and cardiac arrhythmias. Increased risk categories of patients include those with tibial plateau fractures, crush injuries, and prolonged extrication [15]. Femoral neck fractures and ankle fractures are less frequently associated with this complication of orthopedic injury. Pain from passive stretching of the affected compartment is thought to be an early sign, which results in the underutilization of advanced regional anesthesia techniques for otherwise suitable candidates. There are multiple reports attributing a delay in diagnosis in patients receiving regional analgesia, specifically via the subarachnoid and epidural route [22], as well as with patient controlled opioid analgesia. In 2009 Mar et al. published a systematic review in which they analyzed a total of 20 case reports and 8 case series describing compartment syndrome and the effect of analgesia on diagnosis. The majority of these patients received epidural anesthesia (*n* = 23) while peripheral nerve block catheters (*n* = 2) and patient-controlled intravenous analgesia (*n* = 3) were less common. There were no randomized controlled trials or any other outcome-based comparative trials found by the authors. In eight of the case reports reviewed by the authors, pain was present despite postoperative analgesia, but the symptoms were not considered for a prolonged time, resulting in a delay of diagnosis. The authors concluded from their analysis that reports commonly misattribute analgesia as the cause, rather then an association with a delayed diagnosis of compartment syndrome and that all analgesic modalities have been linked to a delay in diagnosis. A high index of suspicion, ongoing patient assessment, and compartment pressure measurement are essential for an early diagnosis of compartment syndrome, independent from the mode of analgesia [23]. 

Recent military experience has not shown any cases of missed compartment syndrome due to effective regional analgesia. A case series review of compartment syndrome occurring in patients receiving peripheral nerve blocks or neuraxial anesthesia reported several early warning signs of this impending complication [23]. The authors concluded that breakthrough pain in spite of previously adequate analgesia and pain in a site unrelated to the injury or surgery warranted a high index of suspicion and close monitoring for compartment syndrome including the use of compartment pressure monitoring. Very similar findings were reported by Cometa et al., who described a case of compartment syndrome while receiving continuous regional analgesia. The patient had complete pain relief from a peripheral nerve block and developed severe pain on the second postoperative day, despite effective nerve block and oral opioid analgesia. Compartment syndrome was diagnosed and treated. The authors came to the conclusion that compartment syndrome can be diagnosed in the presence of effective regional anesthesia and that clinical evaluation and a high index of suspicion are essential in the timely diagnosis [24]. While it is important to recognize the risk of compartment syndrome in this setting and to proceed cautiously, further investigation and collaboration with orthopedists is needed to determine how to better monitor for compartment syndrome, without denying the patient the benefits of regional techniques.

### 3.2. Nerve Injuries and Complications from Regional Techniques

Practitioners involved in the care of acute trauma patients should be aware of potential complications and side effects associated with regional analgesia. These infrequent events include infection, nerve injury, and intravascular injection.

Peripheral nerve injury is a rare complication of regional anesthesia and Auroy et al. reported two cases of nerve injury and one seizure in 11,024 axillary plexus blocks. Out of 3,459 interscalene block procedures, one permanent nerve injury was reported. There were no cardiac arrests, respiratory failures, or deaths reported in 23,784 patients receiving upper-extremity regional nerve block procedures [25]. A prospective study on 257 patients who underwent ultrasound-guided interscalene or supraclavicular nerve blocks did not show any postoperative neurological complications, despite 42 patients in whom an intraneural injection was diagnosed by two blinded anesthesiologist who reviewed the ultrasound images and video offline [26]. 

Local anesthetic toxicity is a concern in all regional anesthesia techniques, but especially when larger volumes of local anesthetic are used. The incidence of this complications is rare and may further be reduced by using low-volume regional anesthesia techniques. O'Donnell et al. were able to demonstrate good pain relief in patients undergoing trauma surgery of the upper extremity when a low volume of local anesthetics was used for axillary brachial plexus blockade [18]. 

Many practitioners are reluctant to perform a regional anesthetic technique in the prehospital setting due to the heightened concern for infection. While many sterile procedures such as chest tube placement and central lines are placed in the field, some believe that it is not worth risking infection by placing a peripheral nerve block in austere conditions, when pain can be treated by alternative means. However, increased opioid administration also has its own risks, including respiratory depression, deep sedation, and the need for airway protection and ventilation during transport.

Reluctance to perform a regional anesthetic technique in the early course of trauma therapy is also influenced by the practitioners fear of nerve damage. Preexisting nerve injury is a relative contraindication for neuraxial techniques and peripheral nerve blocks per American Society of Regional Anesthesia (ASRA) guidelines. The assessment of the extent of injury and neurovascular compromise in the acute trauma patient can frequently be difficult and challenging due to altered mental status as a result of head injury, intoxicants, or sedation. The risk of direct needle trauma to the nerve has decreased with ultrasonography and techniques such as the FICB. While high doses of local anesthetics can be toxic to nerves, clinical concentrations are considered safe [8]. Medicolegal implications are also of concern. The sympathectomy resulting from the placement of a peripheral nerve block increases blood flow to the anesthetized extremity and this may prove beneficial in the presence of vascular compromise in an injured limb. There have been anecdotal reports of successful peripheral nerve blocks in patients with neurovascular compromise, the risks and benefits should be considered on a case-by-case basis. 

Orebaugh et al. looked in a retrospective study at complications from regional anesthesia techniques. The analysis included 5436 consecutive peripheral noncatheter block cases (interscalene, axillary, femoral, sciatic, and popliteal). All procedures where performed by anesthesia staff with or without ultrasound guidance in addition to peripheral nerve stimulation. 3290 procedures were performed with nerve stimulation, but without ultrasound guidance. 2146 procedures were guided by ultrasound and nerve stimulation. A total of eight adverse outcomes (5 seizures and 3 nerve injuries) were recorded in the group without ultrasound guidance and no adverse outcomes in the latter group in which ultrasound guidance was utilized [27]. 

 There was no difference between the two groups in the number of seizures occurring with lower-extremity blocks, or in the frequency of neurologic injury.

Even though the safety of peripheral nerve blocks has improved with the widespread use of ultrasound guidance, the potential risks of local anesthetic toxicity should not be minimized. ASRA and ASA recommend that adequate monitoring capability including pulse oximetry, blood pressure monitoring, and EKG tracing as well as the ready availability of appropriate resuscitation equipment and drugs is essential for the safe performance of regional anesthetic techniques.

### 3.3. The Elderly Patient

There is a paucity of literature about regional anesthesia in elderly patients, especially in the emergency room setting. Beaudoin et al. describes a prospective study in which a convenience sample of 13 patients with a median age of 82 years received an ultrasound-guided femoral nerve block by an emergency medicine physician. The median time to perform the procedure was 8 minutes and there were no complications reported. There was a 44% decrease of pain in pain scores at 15 minutes and 67% decrease at 30 minutes after the nerve block. The authors concluded that ultrasound-guided femoral nerve blocks are feasible to perform in the ED and result in sustained decrease of pain score [28].

### 3.4. Coagulopathy and Anticoagulation

Anticoagulation after surgery is standard practice after surgery and many patients receive anticoagulation or thrombolytics even before surgery. This increases the risk of significant bleeding during regional anesthesia procedures or during catheter removal of continuous peripheral nerve catheters in the postoperative phase. Bickler et al. described significant ecchymoses resulting in delayed hospital discharge in three patients after removal of femoral and sciatic nerve block catheters who received enoxaparin, a low molecular weight heparin [29]. 

The ASRA's Third Consensus Conference on Regional Anesthesia and Anticoagulation recommended using the same guidelines for peripheral regional anesthesia as it is used for neuraxial regional procedures [30]. 

A review of all published cases of clinically significant bleeding or bruising after plexus or peripheral techniques showed in all patients with neurodeficits, neurologic recovery was complete within 6 to 12 months. While bleeding in anticoagulated patients undergoing regional anesthesia may result in significant decreases in hematocrit, the bleeding did not result in irreversible neural ischemia [31]. 

To reduce the risk of complications in anticoagulated patient it is essential to maintain good communication between clinicians and coordinate nerve block procedures and the removal or peripheral nerve block catheters with the dosing schedule of the anticoagulation, avoiding procedures at the peak of anticoagulation.

### 3.5. Availability of Experienced Personnel

The emergency room is a location in which regional anesthesia could be easily and safely performed, but the techniques are underutilized because most emergency medicine physicians are currently not familiar with regional techniques beyond infiltration anesthesia or the block of smaller peripheral nerves. Placement of a continuous catheter for peripheral nerve blockade, nerve plexus blockade, or epidural anesthesia are currently outside the scope of many emergency medicine physicians. 

Few medical or paramedical providers in the prehospital phase possess the adequate level of training and experience to perform these procedures, and the appropriateness of nonphysicians performing these procedures is controversial. The practitioners' lack of expertise in regional anesthesia may cause an unacceptable amount of time to elapse in performing the procedure and delay the treatment of other more serious injuries. The need to consult another practitioner, such as an anesthesiologist, to perform the regional anesthesia procedure can also lead to delayed treatment. Beside increased training of emergency medicine physicians, the availability of anesthesia providers in the emergency room may help to overcome this issue.

## 4. Conclusion

There have been anecdotal reports about regional anesthesia techniques successfully being utilized by European emergency physicians in the field. In Europe, where physicians and often anesthesiologists are utilized in the emergency medicine systems and are brought by ambulance to the scene of an accident, those physicians often utilize their skills and experience of regional techniques in the acute trauma care setting. In addition, the recent experiences of the military have shown promising results in trauma patients with the early use of regional anesthesia, especially continuous catheter techniques, after injury and during transport. It is likely that this experience will be transferred into the civilian sector in the coming years, including continuous catheters for longer-term analgesia. It is important for anesthesiologists to take the lead in adapting regional anesthesia techniques outside of the operating room environment and introduce them into the emergency room and prehospital care setting.
